# Seaweed residue–derived carbon dots composite films for spoilage-responsive monitoring and preservation of large yellow croaker fillets

**DOI:** 10.1016/j.fochx.2026.103626

**Published:** 2026-02-03

**Authors:** Zijia Zhan, Yi Guan, Can Guo, Junchao Huang, Huawei Zheng, Fude Liang, Zhiyu Li, Quan (Sophia) He, Yijing Wu, Qinshan Huang, Jie Yang

**Affiliations:** a Institute of Oceanography, College of Geography and Oceanography, Minjiang University, Fuzhou, China; b Fujian Key Laboratory of Marine Enzyme Engineering, College of Biological Science and Engineering, Fuzhou University, Fuzhou, China; c Department of Orthopedics, Fujian Orthopaedics Research Institute, the First Affiliated Hospital of Fujian Medical University, Fuzhou, China; d Department of Orthopedics, National Regional Medical Center, Binhai Campus of the First Affiliated Hospital, Fujian Medical University, Fuzhou, China; e Fujian Key Laboratory on Conservation and Sustainable Utilization of Marine Biodiversity, Minjiang University, Fuzhou, China; f Department of Engineering, Faculty of Agriculture, Dalhousie University, Truro, NS, Canada

**Keywords:** Chitosan/fish gelatin films, Blueberry anthocyanins, Carbon dots, Intelligent packaging, Aquatic food preservation

## Abstract

A multifunctional packaging film was developed by incorporating blueberry anthocyanins (BA) and seaweed-residue-derived carbon dots (CDs) into a chitosan/fish gelatin (CS/FG) matrix. The CS/FG/BA/0.1CDs film exhibited well-balanced rheology, UV shielding ability, and thermal stability. It also showed improved mechanical strength, enhanced structural uniformity, lower oxygen permeability (4.14 × 10^−11^ g·m/(m^2^·s·Pa)), and superior antioxidant (ABTS: 97.15%; DPPH: 81.88%) and antibacterial activity. Importantly, the film displayed stable and visible colorimetric responses to pH (2−12) and ammonia vapor, enabling real-time freshness monitoring. When applied to large yellow croaker fillets stored at 4 °C, the film effectively delayed spoilage as indicated by reduced pH rise (6.89 ± 0.11), total volatile basic nitrogen content (28.9 ± 1.44 mg N/100 g), and microbial growth after 12 days. Treated samples also retained better texture, water-holding capacity, and sensory scores. These results demonstrate the synergistic effects of BA and CDs in supporting both preservation and intelligent monitoring, offering a sustainable packaging strategy for high-value aquatic products.

## Highlights

• Carbon dots were sustainably derived from *Porphyra haitanensis* residues.
• Carbon dots and anthocyanins were added into chitosan/gelatin hydrocolloid films.
• The fabricated film showed improved physicochemical properties and bioactivities.
• The film exhibited reliable colorimetric response to pH and ammonia vapor.
• Carbon dots and anthocyanins synergistically promoted fish fillet preservation.

## Graphical abstract

**Figure:**
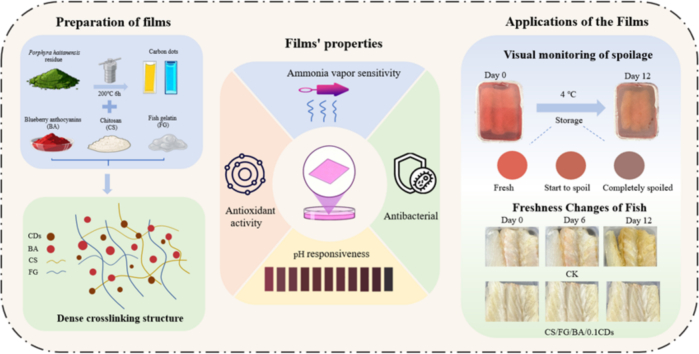

## 1 Introduction

Food packaging plays a vital role in safeguarding food quality and prolonging shelf life by reducing environmental exposure and limiting microbial contamination (Zhu et al., 2025). Plastic material is still commonly used for food packaging, and it has become a major contributor to global environmental pollution (Niu et al., 2025). In addition, the lack of food preservation and real-time monitoring functions weakens the compatibility of traditional packaging with the expectations of today's consumers (Shaikh et al., 2021). Consequently, increasing attention has been directed toward biodegradable intelligent packaging materials that can not only maintain food quality but also provide freshness-related visual information (Sun et al., 2025).

Naturally derived biodegradable polymers such as chitosan and collagen have attracted significant attention as promising alternatives to conventional plastics due to their natural origin, film-forming capacity, and inherent bioactivity. Chitosan is widely recognized for its strong antimicrobial properties, and when processed into films, it provides effective gas barrier performance, thereby enhancing its suitability for use in active food-packaging systems (Sani et al., 2023). Collagen, a structural protein abundant in connective tissues, offers good flexibility, biocompatibility, and mechanical reinforcement (Niu et al., 2025). The film formed by combining the two is excellent in tensile strength and structural stability, thereby rendering it a promising material for sustainable food packaging. However, these films still face several challenges, including limited thermal and ultraviolet resistance, insufficient antioxidant performance, and high sensitivity to moisture (Zheng et al., 2024).

To overcome these limitations, researchers are increasingly focusing on emerging functional materials such as carbon dots (CDs) to enhance film properties (Deepika et al., 2023; Khan, et al., 2026). As zero-dimensional carbonaceous nanomaterials with sizes generally below 10 nm (Luan et al., 2025), CDs, which are synthesized from renewable feedstocks such as agricultural residues (Ghosh et al., 2021), are characterized by low toxicity, excellent water dispersibility, tunable surface chemistry, and favorable biocompatibility. Numerous studies have demonstrated their strong antioxidant and antimicrobial activities (Chen et al., 2023), attributed to the presence of diverse surface chemical functionalities (e.g., hydroxyl, carboxyl, and amine). Such groups are able to neutralize free radicals effectively, thereby reducing oxidative stress. Moreover, CDs can facilitate the formation of reactive oxygen species under some circumstances, which further enhances their antibacterial capacity by disrupting bacterial membranes (Liu et al., 2023). Furthermore, incorporating CDs into biopolymer films can impart significant UV-blocking performance, while also improving the matrix's thermal stability and hydrophobicity (Wang et al., 2022).

As a naturally derived polyphenolic compound and water-soluble colorant, anthocyanins can induce pH-sensitive color changes when incorporated into films, enabling them to function as visual indicators of food freshness (Becerril et al., 2021), particularly in monitoring the quality of high-protein foods such as seafood. During the spoilage of protein-rich foods, volatile basic nitrogenous compounds are generated and released, leading to an increase in the pH of the internal packaging environment (Remedio & Parada Quinayá, 2024). Anthocyanins then undergo distinct color changes in response to volatile basic nitrogenous compounds and pH changes, enabling visual and real-time monitoring of food freshness (Zhang et al., 2025). Unfortunately, no research attempt has been made to integrate anthocyanins and CDs into the biodegradable packaging films for seafood preservation and real-time quality monitoring, despite the synergistic interactions between anthocyanins and CDs might profoundly improve seafood preservation efficiency (Huang et al., 2025).

*Larimichthys crocea*, widely referred to as large yellow croaker, is an important aquaculture species in Asia but is highly susceptible to quality deterioration during storage (Li et al., 2023). With annual production in China exceeding 250,000 tons (China Agricultural Press, 2023), ensuring product quality through efficient preservation and freshness monitoring is crucial. Accordingly, this study aimed to evaluate the performance of a biodegradable composite films incorporating CDs and anthocyanins, with large yellow croaker selected as the research model.

Marine biomass by-products generated during protein extraction are often underutilized despite their rich carbon and heteroatom content, highlighting the need for value-added conversion strategies (Amir et al., 2025). CDs derived from marine macroalgae residues have attracted increasing attention due to their tunable surface chemistry, optical properties, and potential bioactivity, which are closely related to food preservation applications (Luan et al., 2025). Meanwhile, conventional biodegradable films based on chitosan and fish gelatin provide good film-forming ability but suffer from limited UV-blocking capacity and insufficient antioxidant and antibacterial performance (Hadavifar et al., 2025; Uranga et al., 2019). Blueberry anthocyanins (BA) offer natural UV absorption and color responsiveness; however, their instability and limited spectral coverage restrict their standalone effectiveness (Lan et al., 2023). We therefore hypothesized that integrating marine biomass–derived CDs with BA into a chitosan/fish gelatin matrix could yield a multifunctional biodegradable film with enhanced UV shielding, antioxidant and antibacterial properties, while enabling real-time freshness monitoring. Based on this rationale, CDs were synthesized from *Porphyra haitanensis* residues and systematically characterized. Composite films containing BA and varying CDs concentrations were then fabricated and evaluated for physicochemical properties and their preservation performance in large yellow croaker fillets. Therefore, this study aims to explore the feasibility of utilizing marine by-products to develop multifunctional packaging films that integrate preservation and intelligent freshness-monitoring functions.

## 2 Materials and methods

### 2.1 Materials

Chitosan (degree of deacetylation 80–95%, viscosity 50–800 mPa·s) was obtained from Sinopharm Chemical Reagent Co., Ltd. (Shanghai, China).Fish gelatin (purity ≥90%) was sourced from Macklin Biochemical Co., Ltd. (Shanghai, China).Blueberry anthocyanins (purity ≥95%, CAS No. 13306–05-3) were supplied by Yuanye Bio-Technology Co., Ltd. (Shanghai, China).Carbon dots (CDs) were synthesized in the laboratory using *Porphyra haitanensis* residues via a hydrothermal method, as described in Section 2.2. Trisodium citrate (≥ 98% purity, CAS No. 68–04 2) was purchased from Macklin Biochemical Co., Ltd. (Shanghai, China). Glycerol, sodium chloride, reduced iron, and hydrochloric acid were obtained from Chengdu Kelon Chemical Reagent Co., Ltd. (Chengdu, China). Handy plate® Aerobic Count Plate (HP001, 20 tests/pack; Guangzhou Huankai Microbial Sci. & Tech. Co., Ltd., Guangzhou, China). All chemicals were used as received. A total of 80 fresh large yellow croakers (*Larimichthys crocea*, average weight 400–450 g) were sourced from an aquaculture farm in Ningde, Fujian Province, China. The fish were delivered to the laboratory and immediately processed upon arrival.

### 2.2 Preparation and characterization of CDs

Carbon dots (CDs) were synthesized from *Porphyra haitanensis* residues using a hydrothermal method with slight modifications from the procedure reported by Luan et al. (2025). Briefly, 1.0 g of dried *Porphyra haitanensis* residue was dispersed in 20 mL of distilled water and transferred into a 50 mL polytetrafluoroethylene (Teflon)-lined stainless-steel autoclave. The hydrothermal reaction was conducted at 200 °C for 6 h. After cooling to room temperature, the resulting mixture was centrifuged at 12,000 rpm for 10 min, and the supernatant was filtered through a 0.22 μm microporous membrane to remove larger particles and aggregates. The obtained CDs solution was collected and stored at 4 °C in the dark for subsequent characterization and film preparation.

The morphology of CDs was examined by high-resolution transmission electron microscopy (TEM; FEI Talos F200S, Thermo Fisher Scientific, USA) operated at 200 kV. Particle-size statistics were obtained by measuring ≥100 particles from representative TEM micrographs. Surface elemental composition and chemical states were analyzed by X–ray photoelectron spectroscopy (XPS; Escalab 250Xi, Thermo Fisher Scientific, Waltham, USA). Fourier transform infrared (FTIR) spectra were recorded on a Nicolet 6700 spectrometer (Thermo Electron Co., Waltham, MA, USA) over 500–4000 cm^−1^ with 32 scans per spectrum. Ultraviolet–visible (UV–Vis) absorption spectra were collected using a Cary 60 spectrophotometer (Agilent, Santa Clara, USA). Photoluminescence (PL) spectra were acquired on an RF-6700 fluorescence spectrometer (Shimadzu, Tokyo, Japan) with a 1 nm data interval and a scan rate of 6000 nm·min^−1^. All measurements were performed at ambient temperature.

### 2.3 Preparation of composite films

Five types of films were prepared: CS/FG, CS/FG/BA, CS/FG/BA/0.05CDs, CS/FG/BA/0.1CDs, and CS/FG/BA/0.5CDs. For the CS/FG and CS/FG/BA films, chitosan (CS, 2 g) was dissolved in 100 mL of 1% (v/v) acetic acid solution, while fish gelatin (FG, 1 g) was dissolved in 25 mL of distilled water. The two solutions were mixed and stirred at 200 rpm for 30 min. Sodium citrate (0.1 g) and glycerol (0.09 g) were then added to the mixture; for the CS/FG/BA film, blueberry anthocyanins (BA, 2 wt%) were additionally incorporated. For the CS/FG/BA/CDs films, the procedure was similar, except that CS (2 g) was dissolved in 100 mL of carbon dot (CDs) dispersions at different concentrations (0.05%, 0.1%, and 0.5%, w/v), each containing 1% (v/v) acetic acid. The FG solution (1 g in 25 mL distilled water) was then added, followed by BA, sodium citrate, and glycerol. All mixtures were stirred for an additional 6 h, adjusted to pH 3.0 with HCl, and the film-forming solution was left to stand for 6 h under ambient conditions for natural degassing prior to casting. A 30 mL aliquot of each film-forming solution was cast onto plastic Petri dishes and dried at 30 °C for 36 h. The dried films were peeled off and conditioned at 25 ± 1 °C and 55 ± 2% RH for 48 h prior to testing.

#### 2.3.1 Characterization of composite films

##### 2.3.1.1 Scanning electron microscopy (SEM)

The surface morphology of the films was examined via scanning electron microscopy (SEM; Axia ChemiSEM, Thermo Fisher Scientific, USA) equipped with a standard integrated Everhart-Thornley detector (ETD). Initially, the specimens were mounted onto conductive adhesive stubs and sputter-coated with gold for 120 s. Imaging was performed at an accelerating voltage of 15 kV with a consistent magnification of 25,000 × .

##### 2.3.1.2 Viscosity measurement of film-forming solutions

Viscosity measurements of the film-forming solutions were performed on a rotational rheometer (HR-20, Anton Paar, Graz, Austria) using a 40 mm parallel plate geometry. The tests were carried out at 25 °C with a 1 mm gap, and flow curves were obtained over a shear rate interval of 0.1–1000 s^−1^.

##### 2.3.1.3 FTIR and thermal stability analysis

Fourier transform infrared (FTIR) spectra of the composite films were collected using a Nicolet iS50 spectrometer (Thermo Fisher Scientific, Waltham, MA, USA) to identify characteristic functional groups. Measurements were performed in the range of 400–4000 cm^−1^ with a resolution of 4 cm^−1^, and each spectrum was averaged over 64 scans.

Thermal stability was assessed with a TGA 8000 thermogravimetric analyzer (PerkinElmer, USA). Approximately 3 mg of each film was weighed into an alumina crucible and heated from 30 °C to 600 °C at 20 °C/min under a nitrogen flow of 20 mL/min. Both thermogravimetric (TG) and derivative thermogravimetric (DTG) curves were obtained as a function of temperature.

##### 2.3.1.4 Thermal stability analysis

Thermal stability of the various films was assessed utilizing a PerkinElmer TGA 8000 system (USA). Approximately 3 mg of each sample was placed in an alumina crucible and heated from 30 °C to 600 °C at a heating rate of 20 °C/min under a nitrogen atmosphere with a purge flow rate of 20 mL/min. The resulting TG and DTG curves were utilized to analyze the thermal decomposition stages of the different samples.

##### 2.3.1.5 Light-shielding ability and water contact angle (WCA)

The optical transmittance of the films was determined using a UV–Vis spectrophotometer (EVOLUTION Pro, Thermo Scientific, USA). Rectangular strips (40 mm × 10 mm) were cut from each film, and spectra were recorded over 200–800 nm.

Water contact angle (WCA) was measured using a Theta optical tensiometer (OCA20, Dataphysics Instruments, Filderstadt, Germany). A 5 μL droplet of deionized water was placed onto the film surface, and the contact angle was recorded immediately after deposition.

##### 2.3.1.6 Mechanical testing

Mechanical properties of the films were evaluated using a texture analyzer (AG-IC 50 kN, Shimadzu, Tokyo, Japan). Film specimens were cut into strips (50 mm × 10 mm) and clamped with an initial gauge length of 30 mm. Tensile tests were carried out at a crosshead speed of 10 mm/min until rupture, and stress–strain curves were recorded throughout the test.

##### 2.3.1.7 Oxygen permeability (OP) and water vapor permeability (WVP)

Oxygen permeability (OP) and water vapor permeability (WVP)of the films were evaluated at room temperature following a modified method based on Ren et al. (2022). For OP determination, 5 g of an oxygen absorber mixture (iron powder: activated carbon: sodium chloride = 0.5:1.0:1.5, w/w/w) was placed in the bottle (40 mm opening), which was then sealed with the film specimen. The bottle was stored in a desiccator at 25 °C. The decrease in weight of the oxygen absorber was measured after 48 h.

For WVP analysis, 3 g of anhydrous calcium chloride was placed in a brown glass bottle (40 mm opening), and the film specimen was tightly sealed over the mouth of the bottle. The assembly was then kept in a desiccator at 25 °C and 75% relative humidity, controlled by saturated sodium chloride solution. After 48 h, the weight gain of the bottle was determined. All measurements were performed in triplicate, and average values were reported. The WVP and OP were obtained according to the following equation:

(1) $$\mathrm{OP}/\mathrm{WVP}=\frac{\mathrm{\Delta M}\times D}{T\times A\times \mathrm{\Delta P}}\%$$

where: *ΔM* (g) represents the mass change of the bottle, *D* (m) is the average thickness of films, *A* (m^2^) is effective film area, *T* (s) denotes the test time, and *ΔP* (Pa) denotes the partial pressure difference across the both sides of the films (3.1671 × 0.7 kPa at 25 °C).

##### 2.3.1.8 Antioxidant and antibacterial activities

The antioxidant capacity of the films was assessed using DPPH and ABTS radical scavenging assays, following the method of Luan et al. (2025) with slight modifications. Two milligrams of the film samples were added to 3 mL of DPPH or ABTS solution and incubated at room temperature in the dark for 12 h and 6 h, respectively. After incubation, absorbance was recorded at 517 nm for DPPH and 734 nm for ABTS using a full-spectrum microplate reader (ReadMax 1900, Shanghai Shanpu Biotechnology Co., Ltd., China). The radical scavenging activity was calculated using the following equation:

(2) $$\begin{array}{c} \text{Free radical inhibition}\;\left(\%\right)=\left(1-\frac{A_{s}}{A_{c}}\right)\times 100\% \end{array}$$

where *A*_*S*_ was the absorbance of the sample and *A*_*C*_ was the absorbance of the control of DPPH or ABTS solution.

The antibacterial activity of the film samples against Gram-negative *E. coli* and Gram-positive *S. aureus* was evaluated according to a reported method with slight modifications (Guo et al., 2024a). The bacterial strains were cultured in Luria–Bertani (LB) broth at 37 °C for 24 h. The bacterial suspension was then diluted with LB medium to approximately 1.0 × 10^6^ CFU/mL. An aliquot of 100 μL of the suspension was added to a 96-well plate and mixed with 50 μL of the film solution. For the control, 100 μL of the bacterial suspension was combined with 50 μL of sterile distilled water. After incubation at 37 °C for 24 h, bacterial growth was quantified by measuring the absorbance at 600 nm using a microplate reader. The antibacterial rate was calculated as follows:

(3) $$\begin{array}{c} \mathrm{Antibacterial rate}\;\left(\%\right)=\left(1-\frac{A_{t}}{A_{c}}\right)\times 100\% \end{array}$$

where *A*_*t*_ and *A*_*C*_  represent the absorbance values of the treatment and control groups, respectively.

##### 2.3.1.9 pH responsiveness and ammonia vapor responsiveness of films

The colorimetric performance of the films was evaluated according to the method of Hadavifar et al. (2025) with slight modifications. A series of pH-adjusted solutions (pH 2.0 to 12.0) was prepared by adjusting distilled water with 6 mol/L HCl and 6 mol/L NaOH while continuously monitoring the pH with a calibrated pH meter (FiveEasy Plus FE28, Mettler-Toledo Instruments Co., Ltd., Shanghai, China) to investigate the pH sensitivity of the films. Film specimens (2 cm × 2 cm) were placed in Petri dishes (60 mm in diameter) containing the corresponding buffer solution and incubated for 3 min. The color parameters (L*, a*, b*) of the films were then measured using a ColorReader CR8 spectrophotometer (Shenzhen ThreeNH Technology Co., Ltd., Shenzhen, China). The measurements were conducted under D/8 geometry and SCI mode, utilizing a D65 illuminant and a 10° observer angle with an Ф8 mm aperture. Each sample was measured at five random positions to obtain average values. In addition, the obtained L*, a*, and b* values were entered into a digital colorimetric transformation platform (ColorHexa) to generate corresponding color images, providing a direct illustration of the film color changes under different pH conditions.

The NH₃ sensitivity of the films was evaluated according to the method of Chen et al. (2020) with slight modifications. Film specimens (1 cm × 1 cm) were fixed on the top of a transparent Petri dish containing 20 mL of 5% (v/v) ammonium hydroxide solution and maintained at room temperature. During NH₃ exposure, the color changes of the films were recorded every 2 min using a smartphone (iPhone 14 Pro, Apple Inc., USA) under constant LED lighting (approx. 5500 K, 500 Lux) in a light-controlled environment. To ensure high-quality imaging and reproducibility, the camera was fixed on a tripod at a vertical distance of 15 cm with the parameters locked at an aperture of *f*/1.78, ISO 200, shutter speed of 1/60 s, equivalent focal length of 48 mm, and + 0.3 EV exposure compensation. Identical shooting parameters were applied to eliminate the influence of shadows, glare, and ambient light fluctuations on the responsiveness assessment.

### 2.4 Packaging applications for fresh fish fillets

Fish fillets were uniformly cut into portions (20 ± 2 g) and randomly assigned to the different treatment groups (control and various composite film groups) to ensure that any inherent biological variability among individual fillets was incorporated into the random error term. Three fillet cuts were placed in each food-grade plastic box (internal dimensions: 9.0 × 6.5 × 3.8 cm) and sealed with different films: control (CK, commercial food-grade polyethylene film, Miojie, China; thickness: 0.016 mm; WVP: 1.30 × 10^−12^ g·m/(m^2^·s·Pa); OP: 3.23 × 10^−13^ g·m/(m^2^·s·Pa)), CS/FG film, CS/FG/BA film, and CS/FG/BA/0.1CDs film. The boxes were covered with the experimental films and sealed tightly with Parafilm (Bemis, USA) to maintain an airtight environment (**Fig. S1**). All samples were stored at 4 ± 1 °C for 12 days, and analyses were conducted on days 0, 3, 6, 9, and 12. At each sampling time, photographs were taken under identical lighting and camera settings as those described in Section 2.3.1.9 to record both the changes in fish fillet appearance and the color variations of the packaging films during storage. The quality indices included pH, total volatile basic nitrogen (TVB–N), total viable counts (TVC), thiobarbituric acid reactive substances (TBARS), drip loss, texture profile analysis (TPA), and sensory evaluation, were all performed in triplicate (n = 3) to ensure the reliability of the results.

The freshness of large yellow croaker fillets was evaluated by measuring pH, TVB-N, and TVC. The freshness indicators were determined following the methods described by Fan et al. (2009) and Xiao et al. (2023) with slight modifications. For pH, 5.0 g of homogenized meat was mixed with distilled water to a final volume of 50 mL, soaked for 30 min, and the filtrate was measured using a calibrated digital pH meter (FiveEasy Plus FE28, Mettler-Toledo Instruments Co., Ltd., Shanghai, China) equipped with an LE438 pH probe. TVB-N was determined using an automatic Kjeldahl analyzer: a 5.00 g sample was mixed with 0.25 g of MgO and distilled, with the volatile nitrogen absorbed in 2% boric acid and titrated online with 0.01 mol/L HCl. For TVC, 25 g of fish meat was homogenized with 225 mL of sterile saline, and serial 10-fold dilutions were inoculated onto HandyPlate® aerobic count plates and incubated at 36 ± 1 °C for 48 ± 2 h. Results were expressed as average pH, mg N/100 g for TVB-N, and log CFU/g for TVC based on at least duplicate measurements. MDA levels were quantified using the TBARS assay, in which samples were reacted with TCA–TBA reagent, heated, centrifuged, and measured at 532 nm. Results were expressed as mg MDA/kg. Texture properties of fish fillets were measured according to the method of Hadavifar et al. (2025) with slight modifications, using a texture analyzer (AMETEK Brookfield, USA). Samples were cut into rectangular pieces of 2 cm × 2 cm × 3 cm, and each sample was tested five times. The test conditions were as follows: TPA mode with a P/5 cylindrical probe, double compression to 50% deformation, 5 s interval between two compressions, pre-test speed 1 mm/s, test speed 1 mm/s, post-test speed 1 mm/s, and trigger force 5 g.

Drip loss was determined according to the method of Ismail et al. (2023) with slight modifications. The initial weight of each fish fillet was recorded. After storage for the designated period, samples were removed, and surface exudates were gently blotted with filter paper before reweighing. Drip loss was calculated using the following equation and expressed as a percentage:

(4) $$\begin{array}{c} \mathrm{Drip loss}\;\left(\%\right)=\frac{W_{0}-W_{1}}{W_{0}}\times 100\% \end{array}$$

where *W*_*0*_ and *W*_*1*_ represent the weights before and after storage, respectively.

Sensory evaluation was performed by a trained panel of ten graduate students (five males and five females) recruited from the Department of Food Science, all of whom reported no known food allergies or anosmia. The panelists underwent three two-hour training sessions using both fresh and deteriorated fish samples to calibrate their assessments for odor, color, and texture. Following the training, the panelists participated in five formal testing sessions on Days 0, 3, 6, 9, and 12, with each session lasting approximately 30 min. In each session, three replicates of fish fillet samples (approx. 20 g each) were randomly presented. The formal testing sessions were conducted in individual sensory booths under standardized white lighting at a controlled temperature of 22 ± 1 °C in accordance with international sensory laboratory standards. Detailed criteria are listed in **Table S1**, and triplicate measurements were averaged.

### 2.5 Statistical analysis

Statistical analysis was conducted using Minitab 20 (Minitab Inc., USA). Three distinct experiments (*n* = 3) using the same type of film were conducted, and data are expressed as the mean ± standard deviation. One-way ANOVA with Tukey's test was employed for the analyses presented in Section 3.2 to compare the properties of different films. For the evaluation of spoilage-responsive monitoring and preservation effects over time (Section 3.4), a two-way ANOVA was performed to determine the main effects of film type, storage time, and their interaction. Replication was treated as a source of experimental error rather than an independent factor. In all cases, *p* < 0.05 was considered statistically significant.

## 3 Results and discussion

### 3.1 Physicochemical characterization of CDs

To comprehensively investigate the microstructure and physicochemical properties of CDs, various characterization techniques were employed, including TEM, FTIR, XPS, UV–Vis, and PL spectroscopy as presented below.

#### 3.1.1 Morphology and particle size distribution

**Fig. 1:**
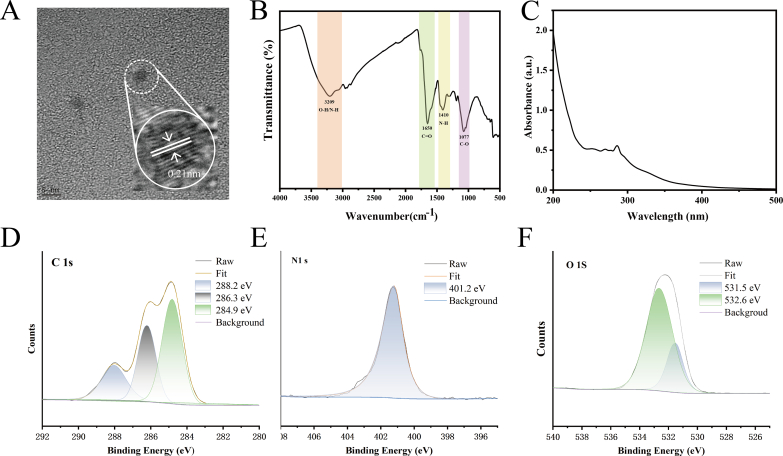
(A) Transmission electron microscopy (TEM) image of carbon dots (CDs) derived from *Porphyra haitanensis* residues; scale bar: 5 nm. (B) Fourier transform infrared (FTIR) spectrum of CDs. (C) Ultraviolet–visible (UV–Vis) absorption spectrum. (D—F) High-resolution X-ray photoelectron spectroscopy (XPS) spectra of carbon (C 1 s), nitrogen (N 1 s), and oxygen (O 1 s), respectively.

TEM characterization results showed that the prepared CDs were roughly spherical, without aggregation **(Fig. S2A)**. The particle size distribution (**Fig. S2B**) ranged from 1 to 3 nm, with an average diameter of approximately 2.27 nm. High-resolution TEM observations showed clear lattice fringes with a measured interplanar distance of 0.21 nm **(**Fig. 1A**)**, which serves as a direct indicator of highly ordered carbon atoms within the CDs, confirming the presence of crystalline carbon domains (Yin et al., 2023). These structural features provide the basis for the CDs' stable optical properties and robust functional activities in food preservation (Navaneethan et al., 2025).

#### 3.1.2 Fourier transform infrared (FTIR) analysis

The FTIR spectrum (Fig. 1B) indicated that the surface of the CDs is rich in hydrophilic and polar functional groups. Specifically, a broad band at ∼3209 cm^−1^ was attributed to O—H and/or N—H stretching vibrations (Navaneethan et al., 2025). The absorption band near ∼1650 cm^−1^ corresponds to the C=O stretching vibration of carbonyl groups (Yang et al., 2014). Additionally, the peaks at 1410 cm^−1^ and 1077 cm^−1^ are generally linked to the stretching of C—O and C—N bonds, respectively (Li et al., 2022). The presence of these hydrophilic groups is likely to improve the aqueous compatibility of CDs, facilitating their uniform dispersion and interfacial interaction in hydrophilic matrices (Tang et al., 2025).

#### 3.1.3 Ultraviolet–visible spectroscopy (UV–vis) and photoluminescence spectra

The UV–Vis absorption spectrum (Fig. 1C) exhibited a broad absorption band in the range of 200–400 nm. A distinct peak around 286 nm is attributed to the n–π* transition of C=O groups (Durairaj et al., 2022). These findings indicated the coexistence of conjugated π-systems and oxygen-containing functional groups in the CDs structure. The ability of CDs to absorb UV light suggests their potential to enhance the UV-blocking efficiency of composite films, thereby extending the shelf life of packaged food products. The photoluminescence (PL) spectrum (**Fig. S3**) demonstrated that the CDs exhibited typical excitation-dependent fluorescence behavior. Under excitation at 364 nm, the CDs exhibited a strong blue emission peak at 447 nm. This excitation-dependent emission behavior is generally associated with the presence of multiple emissive centers or surface trap states (Chen et al., 2018). Meanwhile, the surface chemical groups reflected in the PL behavior can also contribute to functional performance, such as antioxidant activity (by helping quench reactive radicals) and antibacterial support (by interfering with microbial cells) (Hadavifar et al., 2025; Luan et al., 2025). Therefore, UV–vis and PL do not only “identify” the CDs; they provide qualitative evidence of the surface characteristics that underpin their functional roles.

#### 3.1.4 X-ray photoelectron spectroscopy (XPS) analysis

The surface elemental composition and chemical states of the CDs were further analyzed by XPS. The spectrum (**Fig. S4**) showed that the major elements present were carbon (C, 285.0 eV), nitrogen (N, 399.5 eV), and oxygen (O, 532.3 eV). The high-resolution C 1 s spectrum (Fig. 1D) could be deconvoluted into four characteristic peaks at 284.9 eV (C–C/C=C), 286.3 eV (C—O), 288.2 eV (C=O), and 288.8 eV (N–C=N) (Chowdhury & Kumar Das, 2021). Notably, the dominant peak at 284.9 eV in Fig. 1D indicated that graphitized carbon was the predominant structural component. The N 1 s spectrum (Fig. 1E) displayed a single peak at 401.2 eV, attributed to –NH_2_ groups, indicating that nitrogen was primarily incorporated in the form of amines (Beamson & Briggs, 1993; Hadavifar et al., 2025). The O 1 s spectrum (Fig. 1F**)** showed peaks at 531.5 eV and 532.6 eV, corresponding to C=O and C–O/–OH groups, respectively (Yin et al., 2023). The dominant peak at 532.6 eV further confirmed the presence of hydroxyl and carboxyl-containing polar groups, consistent with FTIR results. In summary, these CDs possess a partially graphitized core and a surface rich in oxygen/nitrogen-containing functional groups, which are intrinsically linked to their multi-functionality. Specifically, the hydroxyl and carboxyl groups facilitate excellent aqueous dispersibility and antioxidant activity through hydrogen bonding and electron donation (Xiang et al., 2025). The carbonyl groups significantly enhance UV-blocking performance (Rowell et al., 2022). Furthermore, the amino-containing groups are primarily responsible for the CDs' biocompatibility and potential antibacterial efficacy (Younes & Rinaudo, 2015). These structural attributes are expected to enhance the interfacial interaction, rheological behavior, and functional properties of the composite films, as discussed in the subsequent sections.

### 3.2 Characterization of composite films

#### 3.2.1 Scanning electron microscopy (SEM) analysis

**Fig. 2:**
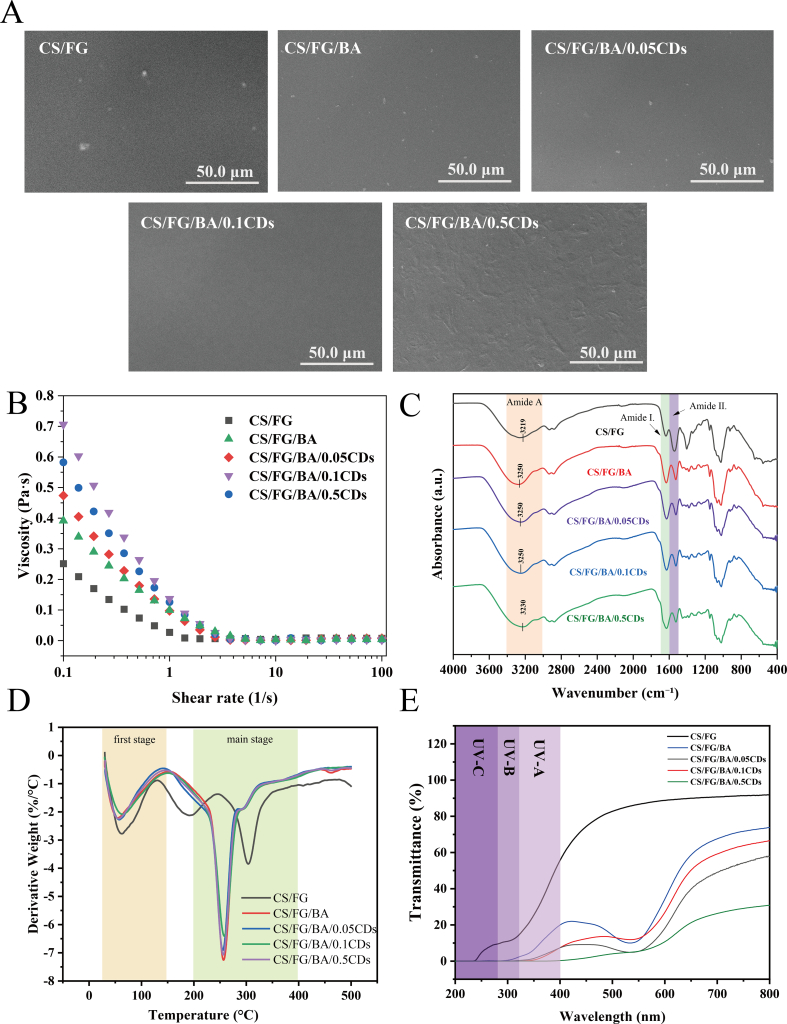
(A) Scanning electron microscopy (SEM) images of the surface morphology of composite films. (B) Rheological characterization of film-forming solutions. (C) Fourier transform infrared (FTIR) spectra of the films. (D) Derivative thermogravimetric (DTG) curves of the films. (E) Ultraviolet–visible (UV–Vis) transmittance spectra of the films. Film formulations: CS/FG: chitosan/fish gelatin film; CS/FG/BA: chitosan/fish gelatin film containing blueberry anthocyanins (BA);

SEM was used to assess the films' uniformity and smoothness, as shown in Fig. 2A. The CS/FG film displayed a dense and continuous structure with minor surface protrusions observed, which might have resulted from localized gelatin aggregation or uneven shrinkage during drying, indicating slight microphase separation between CS and FG. The addition of BA did not alter the film morphology, suggesting its good compatibility and uniform dispersion with the CS/FG matrix. This aligns with findings by Wagh et al. (2023), where polyphenol incorporation maintained film uniformity due to strong molecular affinity. With 0.05% CDs, the film remained smooth and compact, indicating uniform CD dispersion. At 0.1% CDs, the surface smoothness was further improved, attributed to the favorable compatibility, uniform distribution, and effective incorporation of CDs into the interspaces of the polymer matrix. However, at 0.5% CDs, surface irregularities and slight wrinkling appeared, implying CD aggregation and reduced compatibility. Excessive CDs has been shown to disrupt the polymer network continuity and compromise functional properties (Hadavifar et al., 2025).

#### 3.2.2 Rheological behavior of the film-forming solutions

The rheological behavior of the film-forming solutions **(**Fig. 2B**)** directly influenced the final film quality. All samples exhibited pronounced shear-thinning behavior, as viscosity decreased markedly with increasing shear rate, confirming that the solutions were typical non-Newtonian fluids (Rao, 2014). This phenomenon was mainly attributed to the alignment and partial disentanglement of polymer chains under shearing, which reduces internal resistance (Ata et al., 2025). Relative to the CS/FG film, the viscosity of the CS/FG/BA film exhibited a notable increase, likely due to non-covalent interactions—such as hydrogen bonding and π–π stacking—between BA and the polymeric matrix, which enhanced molecular entanglement and stabilized the network structure (Homayoonfal et al., 2022). Upon incorporation of CDs, the viscosity of the film-forming solutions displayed a non-monotonic trend, initially increasing and then decreasing with rising CDs content **(**Fig. 2B**)**. The CS/FG/BA/0.1CDs sample showed the highest viscosity, which was ascribed to the physical filling effect of well-dispersed nanoparticles that restrict polymer chain mobility. However, when the CDs concentration reached 0.5% (CS/FG/BA/0.5CDs), the viscosity decreased. This reduction is mainly attributed to particle-particle aggregation at excessive loadings, which disrupted homogeneous dispersion and diminished the effective contribution of CDs to the bulk viscosity. Similar non-linear rheological responses have been widely reported in polymer–nanofiller systems, where optimal dispersion enhances viscosity whereas overloading leads to aggregation and reduced viscoelasticity (Wang et al., 2023).

#### 3.2.3 FTIR analysis of films

FTIR spectroscopy **(**Fig. 2C**)** was employed to identify the functional groups present in the composite films. For the pristine CS/FG film, the characteristic absorption bands appeared mainly at 1600–1700 cm^−1^ (amide I, corresponding to C=O stretching vibrations), 1500–1600 cm^−1^ (amide II, associated with N—H bending vibrations), along with the broad region of 3000–3500 cm^−1^ (amide A), corresponding to O–H/N–H stretching vibrations (Barth, 2007; Coates, 2000). As for the amide I and amide II region, the addition of BA led to an enhancement in the amide I band intensity and a reduction in the amide II band intensity (Muyonga et al., 2004). This spectral change indicates strong intermolecular interactions between BA and the CS/FG matrix, potentially resulting in conformational rearrangements of the biopolymer chains and reorganization of the hydrogen bonding network. These results were in agreement with the findings of Lu et al. (2025), who reported similar FTIR spectral variations upon interaction of polyphenolic compounds with biopolymers. For the CDs addition, amide I band (∼1630 cm^−1^) intensity was enhanced with increasing CD concentration, which was mainly attributed to the CDs' abundant carbonyl (C=O) groups, which exhibit strong characteristic absorption near 1630 cm^−1^, overlapping with the amide I region of the polymer matrix region as observed in Fig. 1B. As shown in Fig. 2C, after adding BA and CDs, the amide A region showed slight blue-shifts with subtle intensity changes. The amide A blue shift potentially suggested hydrogen-bond reorganization, which could be attributed to the partial disruption of –OH/–NH self-interactions in favor of specific BA/CD–matrix contacts (e.g., C=O···H–N), likely contributing to the formation of a denser, more homogeneous network (Wan & Thompson, 2024).

#### 3.2.4 Thermal stability analysis of films

The thermal stability of the composite films was evaluated via Derivative Thermogravimetry (DTG), as shown in Fig. 2D. The initial weight loss stage (30–150 °C) is primarily attributed to the evaporation of physically adsorbed and bound water, consistent with previous reports (Hashemi et al., 2025). The second mass-loss stage (150–200 °C) is a mid-temperature event that is frequently reported in plasticized chitosan/gelatin-type matrices (Hadavifar et al., 2025). In many such systems, this region is often associated with the release of low-molecular-weight species (e.g., plasticizer-related loss and/or other small components) and early structural changes of the biopolymer network (Vieira et al., 2011). In the pristine CS/FG film, a distinct DTG peak appeared near 180 °C, indicating a faster mass-loss process in this temperature window. Notably, this peak was greatly reduced in the BA/CDs-modified films, suggesting that the additives altered the way small molecules and polymer chains behave upon heating. This change is consistent with strengthened overall association within the matrix (e.g., increased intermolecular interactions), which can reduce the mobility of low-molecular-weight species and make the thermal response in this region less pronounced (Abdullah et al., 2022). The main degradation of CS/FG/BA appeared between 200 and 300 °C as presented in Fig. 2D, in which the degradation rate was decreased with the increase of CDs concentration, presumably due to the carbonaceous nature of CDs. This speculation was supported by the Thermogravimetric Analysis (TGA) results as illustrated in **Fig. S5**, in which CDs-incorporated films exhibited much higher residual carbon contents compared to the pristine CS/FG film.

#### 3.2.5 Optical transmittance and UV–blocking performance

The optical properties of film materials, particularly their UV–blocking ability, play a crucial role in delaying photo-oxidation processes (Roy et al., 2024). As shown in Fig. 2E, the pure CS/FG film exhibited high transmittance in the UV region (200–400 nm), with noticeable penetration in both the UV–A (320–400 nm) and UV–B (280–320 nm) ranges, indicating limited UV–shielding capacity. This observation agrees with previous findings that unmodified CS films generally exhibit insufficient UV shielding (Shen et al., 2025). With the incorporation of BA, the UV transmittance decreased markedly, especially in the UV–B region, where it dropped to around 3%, demonstrating excellent UV absorption capability. As natural polyphenolic compounds, BA contain phenolic hydroxyl groups and conjugated systems capable of effectively absorbing UV light, thereby imparting light-shielding ability to the films—a phenomenon supported by several studies (Dong et al., 2024). For example, Zhao et al. (2023) found that blood orange-derived anthocyanins almost completely blocked UV irradiation, effectively extending the shelf life of dairy products. Further addition of CDs at different concentrations led to a progressive enhancement in UV–blocking capacity. As the CDs content increased from 0.05% to 0.5%, transmittance across the UV range gradually decreased; notably, the 0.5% CDs film achieved almost complete blocking (transmittance 200–400 nm range), exhibiting outstanding UV–shielding performance. This effect is mainly attributed to the excellent UV absorption of CDs (Riahi et al., 2025), and the n–π* electronic transitions of oxidized functional groups such as carbonyls on their surface, which facilitate UV light absorption and hinder light transmission (Zhou et al., 2017).

#### 3.2.6 Water contact angle (WCA)

**Fig. 3:**
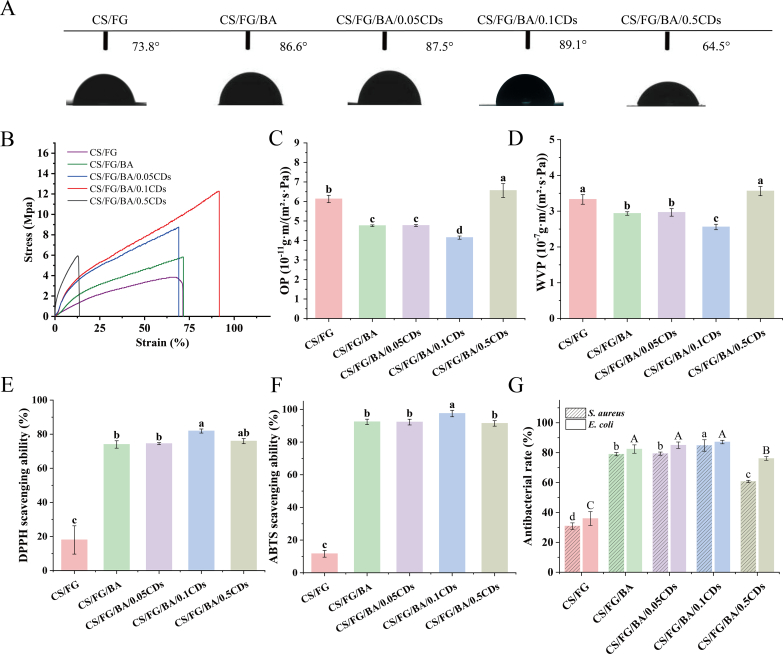
(A) Water contact angles (WCAs) of different composite films. (B) Tensile stress-strain curves. (C) Oxygen permeability (OP). (D) Water vapor permeability (WVP). (E) 2,2-Diphenyl-1-picrylhydrazyl (DPPH) radical scavenging activity. (F) 2,2′-Azino-bis (3-ethylbenzothiazoline-6-sulfonic acid) (ABTS) radical scavenging activity. (G) Antibacterial activity of different films against *S. aureus* and *E. coli*. Data are expressed as mean ± standard deviation (*n* = 3). Statistical significance was determined by one-way ANOVA followed by Tukey's test. Different lowercase letters indicate significant differences among films against *S. aureus* and *E. coli*, respectively (*p* < 0.05). Film formulations: CS/FG: chitosan/fish gelatin film; CS/FG/BA: chitosan/fish gelatin film containing blueberry anthocyanins (BA); CS/FG/BA/0.05CDs: CS/FG/BA film containing 0.05% carbon dots (CDs); CS/FG/BA/0.1CDs and CS/FG/BA/0.5CDs: CS/FG/BA films containing 0.1% and 0.5% CDs, respectively.

The surface wettability of the composite films was evaluated by measuring the WCA (Fig. 3A). Adding BA increased the WCA to 86.6°, and incorporating CDs produced a further, concentration-dependent increase (87.5° for CS/FG/BA/0.05CDs and 89.1° for CS/FG/BA/0.1CDs), indicating enhanced surface hydrophobicity (Lim & Shon, 2018). This enhancement is suggested to be associated with the incorporation of BA, which is expected to form intermolecular hydrogen bonds and hydrophobic associations with gelatin chains. According to Xu et al. (2025), such interactions can reduce the availability of free hydrophilic groups and promote a denser polymer network, thereby contributing to the improved moisture resistance of the composite films. The further increase in hydrophobicity with CDs incorporation is attributed to synergistic cross-linking between components, resulting in the decreased availability of free hydroxyl groups within the polymer matrix (Wagh et al., 2023). However, when the concentration of CDs increased to 0.5%, the contact angle decreased to 64.5°. This reduction can be attributed to: (1) the aggregation and uneven distribution of CDs at higher loadings, which disrupted the film structure and render it more susceptible to water penetration; (2) the surface of CDs is rich in hydroxyl, carboxyl, and other polar functional groups, which enhanced the film's affinity for water molecules (Ezati et al., 2022).

#### 3.2.7 Mechanical properties

The mechanical integrity of packaging films is crucial during handling, transportation and storage (Abdullah et al., 2022). Tensile tests **(**Fig. 3B**)** showed that all samples displayed a polymer-like response with a nearly linear increase in stress with strain, followed by strain hardening to a maximum stress and final fracture (Hosseini et al., 2013). The CS/FG film exhibited a tensile strength (TS) of 3.8 MPa and an elongation at break (EB) of approximately 67%, providing a solid mechanical foundation for the incorporation of functional components. After the incorporation of BA, the TS slightly increased to 5.8 MPa, while the EB remained around 71%. The increase in TS upon BA incorporation is consistent with previous findings, which suggest that hydrogen bonding between BA and polymer chains strengthens intermolecular cohesion and enhances network compactness (Wang et al., 2019). Both TS and EB increased with 0.05% and 0.1% CDs, and the optimum performance was achieved at 0.1%, where TS reached 12.2 MPa and EB rose to 91.6%. This improvement is attributed to the well-dispersed CDs that filled the intermolecular voids within the network and facilitated stress transfer, resulting in a stronger and more ductile film. In contrast, increasing the CDs content to 0.5% resulted in a sharp reduction in both TS and EB, accompanied by obvious brittle fracture behavior. This concentration-dependent deterioration is likely due to CDs aggregation at higher loadings, which generated micro-defects and discontinuities within the polymer matrix. Similar phenomena have been observed in CS/FG nanocomposite films, where low-level CDs maintained mechanical integrity, whereas excessive filler loading induced aggregation and weakened film strength (Murugan et al., 2024).

#### 3.2.8 Oxygen permeability (OP) and water vapor permeability (WVP)

The pure CS/FG film exhibited an OP of 6.13 × 10^−11^ g·m/(m^2^·s·Pa), as shown in Fig. 3C. Upon incorporation of BA, the OP decreased significantly (4.74 × 10^−11^ g·m/(m^2^·s·Pa)), likely due to the denser packing of polymer chains, which reduced the free volume and hindered oxygen diffusion (Jančič et al., 2021). These observations are consistent with previous studies on BA-containing chitosan films (Silva-Pereira et al., 2015). A further reduction in OP was observed with the addition of CDs, in particular with 0.1% CDs, reaching 4.14 × 10^−11^ g·m/(m^2^·s·Pa), indicating that well-dispersed CDs enhanced the compactness of the polymer matrix. However, at 0.5% CDs loading, the OP values increased again, likely due to structural defects induced by CDs aggregation, which facilitated oxygen permeation (Hanani et al., 2019).

The WVP of the pure CS/FG film was measured at 3.2 × 10^−7^ g·m/(m^2^·s·Pa) **(**Fig. 3D**)**. The addition of BA resulted in a statistically significant change (2.9× 10^−7^ g·m/(m^2^·s·Pa)), likely due to the formation of additional hydrogen bonds between BA and the CS/FG matrix, which reduced the number of free hydrophilic sites available for water vapor transmission (Li et al., 2024). At 0.05% CDs, no significant difference was observed in WVP. Upon the incorporation of 0.1% CDs, WVP reached a lowest value of 2.5 × 10^−7^ g·m/(m^2^·s·Pa). At higher CDs concentrations (0.5%), the WVP increased again, approaching the value observed for the pure CS/FG film, which could be attributed to CDs aggregation disrupting the film's structural integrity (Campalani et al., 2022).

#### 3.2.9 Antioxidant activity

The accumulation of free radicals accelerates food deterioration, therefore, improving the antioxidant properties of packaging film is crucial for delaying food oxidative spoilage (Priyadarshi et al., 2018). The antioxidant properties of the fabricated films were evaluated using 2,2′-azino-bis (3-ethylbenzothiazoline-6-sulfonic acid) (ABTS) and 2,2-diphenyl-1-picrylhydrazyl (DPPH) radical scavenging assays as presented in Fig. 3E**&F**. The pure CS/FG film showed very low scavenging activity (11.61 ± 2.05% for ABTS and 17.98 ± 8.30% for DPPH), indicating limited intrinsic antioxidant capacity. Incorporation of BA markedly enhanced scavenging to 92.36 ± 0.14% (ABTS) and 73.96 ± 2.17% (DPPH), confirming the effectiveness of BA as a natural antioxidant in the film. The BA's multiple hydroxyl groups can stabilize free radicals through hydrogen donation (Liu et al., 2021). The incorporation of 0.05% CDs did not significantly affect the antioxidant activity compared with the CS/FG/BA group. However, a significant increase in antioxidant activity was observed at 0.1% CDs, reaching 97.15 ± 1.98% (ABTS) and 81.88 ± 1.19% (DPPH), likely because CDs are rich in carboxyl and hydroxyl groups, as well as π–π conjugated structures, which can promote electron transfer (Gao et al., 2023; Guo et al., 2024). However, at higher CDs loadings (0.5%), film's antioxidant activity declined slightly, probably due to CDs aggregation that reduced the accessibility of active antioxidant sites.

#### 3.2.10 Antibacterial activity

The antibacterial activity of the films was investigated to evaluate their potential for food preservation. Fig. 3G illustrates the antibacterial activity of the composite films against *S. aureus* (Gram-positive) and *E. coli* (Gram-negative). The pure CS/FG film displayed relatively low antibacterial efficacy, with inhibition rates of 30.68 ± 2.14% against *S. aureus* and 35.92 ± 4.62% against *E. coli*, indicating the limited inherent antibacterial properties of the base polymer matrix.

Upon incorporation of BA, the antibacterial performance was significantly enhanced, achieving 78.94 ± 1.10% inhibition against *S. aureus* and 82.38 ± 2.79% against *E. coli*. Previous studies have shown that the phenolic hydroxyl groups in BA can interfere with intracellular enzyme activity and energy metabolism, thereby effectively suppressing bacterial growth (Jeyaraj et al., 2023). However, the incorporation of 0.05% CDs did not result in a statistically significant improvement in antibacterial activity compared with the CS/FG/BA group, suggesting that a low CDs content was insufficient to inhibit *S. aureus* and *E. coli*.

The incorporation of 0.1% CDs into the CS/FG/BA composite film yielded an antibacterial response against *E. coli* that was statistically comparable to the unmodified CS/FG/BA group. Conversely, a significant enhancement in the inhibition rate was observed against *S. aureus*, reaching 84.79 ± 3.83%. Similar observations were reported by Das et al. (2022), who found that clove-derived CDs exerted strong antibacterial effects against *S. aureus*. These findings underscore the fundamental structural disparity between Gram-positive and Gram-negative bacteria: the absence of an outer membrane in *S. aureus* renders it more vulnerable to BA, whereas the outer membrane in *E. coli* confers greater resistance (Luan et al., 2025). Nonetheless, both strains remained highly susceptible to the modified film.

Increasing the CDs concentration to 0.5% resulted in significantly lower antibacterial activity than the CS/FG/BA and CS/FG/BA/0.1CDs. This reduction might be attributed to the aggregation of CDs at higher loadings, which decreased their effective surface area and weakened their dispersion, thereby lowering the efficiency of bacterial contact and ROS generation.

In summary, after adding BA and different amounts of CDs into the CS/FG matrix, the film-forming solutions showed typical shear-thinning behavior, and no profound alteration in functional groups was observed through FTIR analysis. TGA and UV–Vis transmission tests demonstrated improved thermal stability and UV-blocking ability after BA and CDs addition. With the addition of 0.1% CDs into CS/FG/BA film, the film surface remained moderately hydrophobic, but became distinctly hydrophilic at 0.5% CDs. Mechanically, the presence of 0.1% CDs maintained well balance between TS and EB, while 0.5% CDs made the films brittle. The CS/FG/BA/0.1CDs film also exhibited the best barrier performances, the highest antioxidant activity, along with satisfactory antibacterial activity. In view of the aforementioned characterization results, the CS/FG/BA/0.1CDs film was further investigated for its sensitivity to pH changes and ammonia vapor to assess its feasibility as an intelligent packaging indicator for real-time food freshness monitoring (Hadavifar et al., 2025).

### 3.3 Response of CS/FG/BA/0.1CDs to pH variation and ammonia vapor

#### 3.3.1 Response of CS/FG/BA/0.1CDs to pH variation

**Fig. 4:**
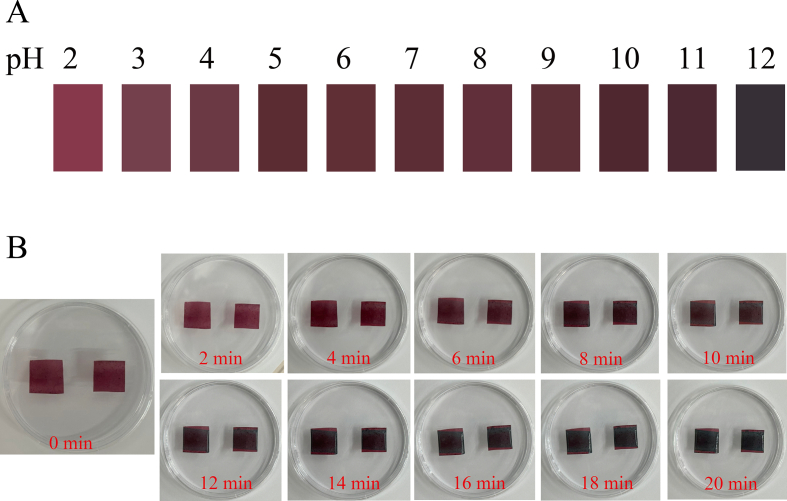
(A) Color change of CS/FG/BA/0.1CDs in different pH solutions. (B) Color response of CS/FG/BA/0.1CDs to ammonia (NH_3_) vapor. Abbreviations: CS, chitosan; FG, fish gelatin; BA, blueberry anthocyanins; CDs, carbon dots from *Porphyra haitanensis* residues. The “0.1” refers to 0.1% (w/v, relative to the matrix) loading of CDs.

To evaluate the potential of the films as intelligent indicators for food freshness monitoring, their color response to pH variation was investigated. As shown in Fig. 4A, the fabricated CS/FG/BA/0.1CDs films exhibited pronounced color response across the pH range of 2–12. Under strongly acidic conditions (pH 2), the films appeared rose-red. With increasing pH, the color progressively darkened, shifting from purplish-red to brownish-purple, and finally to dark violet-black at pH 12. These distinct color variations were easily distinguishable to the naked eye, demonstrating excellent visual response capability. This phenomenon can be attributed to the reversible structural transitions of BA under different pH environments. In acidic conditions, BA predominantly exists as the stable flavylium cations, which exhibits red coloration. Under neutral to alkaline conditions, the flavylium cations gradually hydrolyze into neutral carbinol bases, quinonoidal bases, or salt forms, leading to visible shifts toward brownish-purple. These pH-responsive behaviors of BA pigments have been previously reported by Mu et al. (2025).

#### 3.3.2 Response of CS/FG/BA/0.1CDs to ammonia vapor

Since ammonia (NH₃) is a major gas generated from the spoilage of protein-rich foods during storage, the colorimetric response of the films to NH₃ vapor was further investigated (Abdullah et al., 2022; Hadavifar et al., 2025). The CS/FG/BA/0.1CDs films exhibited a time-dependent color evolution upon exposure to NH₃ vapor, which reflects both the inherent responsiveness of BA and the gradual accumulation of NH₃ vapor volatilized from the 5% ammonia solution, as shown in Fig. 4B. Initially, the films appeared reddish-purple. With prolonged exposure, the color progressively darkened toward purplish-black, and the entire transition was clearly distinguishable by the naked eye within 4 min. These color changes are consistent with the observations made from CS/FG/BA/0.1CDs response to pH variation. When NH₃ vapor interacts with waters within the film, ammonium hydrates (NH_4_OH) can be formed (Lan et al., 2023). The readily available hydroxide ions (OH^−^) then induce structural rearrangements of BA within the film (Chen et al., 2020), thereby altering the visible light absorption spectrum and resulting in a detectable color change (Agunos et al., 2020; Khan et al., 2026). In terms of response behavior, the films exhibited perceptible color variation within 4 min, with the color intensifying significantly by 10 min and reaching an apparent plateau within the 20-min exposure window tested, during which no further visually noticeable changes were observed. This indicated that the films possess high sensitivity and a rapid response rate toward NH_3_ vapor. Similar pH and NH_3_ response were also observed in the CS/FG/BA films **(Fig. S6 & S7)**, indicating that the incorporation of CDs did not alter the inherent color responsiveness of anthocyanins, which remained stable for 4–20 min.

### 3.4 Application in large yellow croaker preservation

#### 3.4.1 Visual indicators during refrigerated storage

**Fig. 5:**
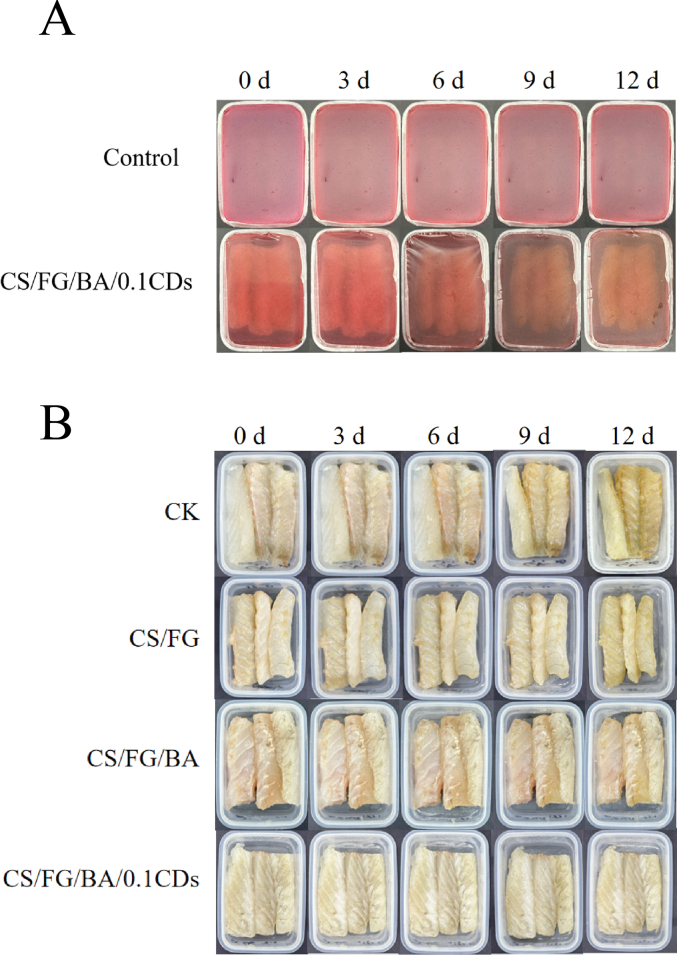
Intelligent monitoring and preservation evaluation of large yellow croaker fillets. (A) Colorimetric response of the CS/FG/BA/0.1CDs films during storage. “Control” denotes the film stored in the same container under identical conditions without fish fillets, to verify color stability in the absence of fish-derived volatiles. (B) Photographs of fish fillets at different storage intervals for visual quality evaluation; the films were removed before imaging to avoid obstruction and enable direct observation of fillet appearance. Film formulations: CK: polyethylene (PE) film wrap; CS/FG: chitosan/fish gelatin film; CS/FG/BA: chitosan/fish gelatin film containing blueberry anthocyanins (BA); CS/FG/BA/0.1CDs: CS/FG/BA film containing 0.1% carbon dots (CDs). (For interpretation of the references to color in this figure legend, the reader is referred to the web version of this article.)

As shown in Fig. 5A, the CS/FG/BA/0.1CDs film exhibited a pronounced color change during refrigerated storage. To determine whether the discoloration was triggered by volatile alkaline compounds released during fish spoilage rather than by oxidation of the films, a blank control group without fish fillet was included. Throughout storage, the control group remained nearly unchanged in color, demonstrating good stability of CS/FG/BA/0.1CDs film under refrigeration. In contrast, the films used to package fish fillet underwent storage time-dependent color changes, turning dark red by day 6 and brownish-purple by day 12. These transitions closely corresponded with the increase in pH level during spoilage, confirming the films' ability to sensitively indicate volatile alkaline accumulation via a pH-responsive mechanism and highlighting their potential as intelligent indicators for seafood storage.

Consistent with these color changes, the external appearance of fish fillets also varied among groups **(**Fig. 5B**)**. The CK group was packaged with polyethylene (PE) film. At day 0, all fillets appeared pale-white, glossy, and firm, indicative of high freshness. By day 3, CK fillets showed slight dullness, whereas composite film-treated groups retained better appearance, with the CS/FG/BA/0.1CDs group showing the least change. At day 6, CK fillets turned yellow with surface water accumulation and tissue loosening, while CS/FG and CS/FG/BA samples displayed moderate changes. In contrast, the CS/FG/BA/0.1CDs group maintained a bright, dry surface and firm texture. By day 9, CK fillets had turned brown with severe exudation and structural damage, CS/FG samples suffered dehydration and loosening, and CS/FG/BA fillets remained relatively intact. Notably, the CS/FG/BA/0.1CDs samples still exhibited minimal changes. At day 12, CK and CS/FG fillets showed severe deterioration, including color darkening, surface mucus accumulation, and tissue collapse, while CS/FG/BA samples exhibited mild deterioration with partial discoloration and surface softening. The CS/FG/BA/0.1CDs group maintained a cleaner appearance, structural integrity, and the least discoloration. Overall, the CS/FG/BA/0.1CDs films not only demonstrated stable and sensitive color responsiveness but also effectively delayed visual deterioration of fish fillets. Their superior preservation efficacy was likely attributed to the synergistic effect induced by BA and CDs addition, underscoring their potential for application in smart preservative packaging.

#### 3.4.2 Changes in pH, TVB–N, TVC, TBARS, and texture

During storage, enzymatic and microbial activity can degrade carbohydrates, proteins, and lipids, producing volatile nitrogenous compounds (Li et al., 2024; Remedio & Parada Quinayá, 2024). Therefore, TVC is widely recognized as a reliable indicator of freshness and overall quality (Prabhakar et al., 2020). The accumulation of TVB–N reflects the degree of protein hydrolysis and spoilage, while the resulting basic nitrogenous compounds can induce pH shifts, which can be effectively monitored using intelligent packaging film (Bakhshizadeh et al., 2023).

**Fig. 6:**
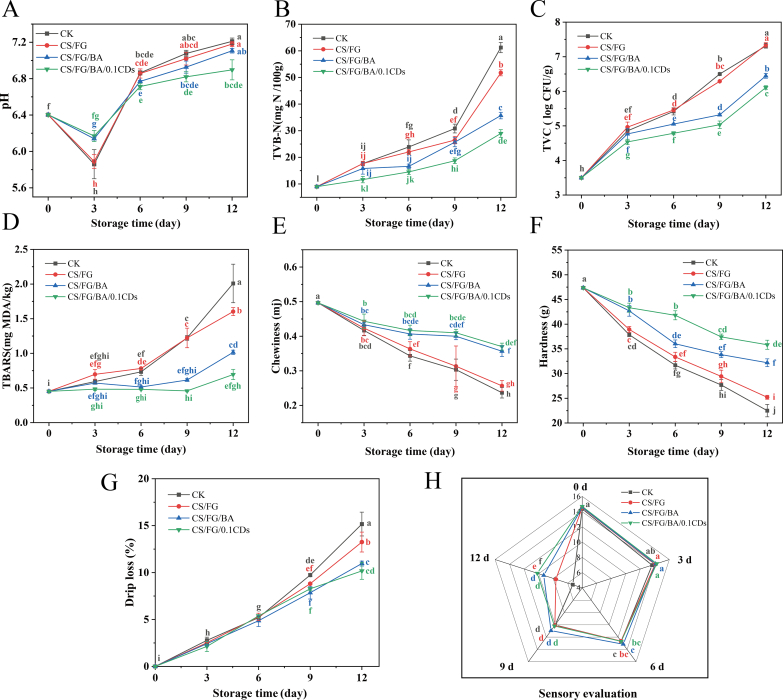
Changes in physicochemical, microbiological, and sensory quality indicators of large yellow croaker fillets during refrigerated storage (4 °C) under different films. (A) pH. (B) Total volatile basic nitrogen (TVB–N, mg N/100 g). (C) Total viable counts (TVC, log CFU/g). (D) Thiobarbituric acid reactive substances (TBARS, mg MDA/kg sample). (E) Chewiness. (F) Hardness. (G) Drip loss. (H) Overall sensory evaluation scores at 0, 3, 6, 9, and 12 days. Data are expressed as mean ± standard deviation (*n* = 3). Statistical significance was evaluated by two-way ANOVA to determine the effects of film type, storage time, and their interaction. Different lowercase letters indicate significant differences (*p* < 0.05) among treatment combinations (film type × storage time). Film formulations: CK: polyethylene (PE) film wrap; CS/FG: chitosan/fish gelatin film; CS/FG/BA: chitosan/fish gelatin film containing blueberry anthocyanins (BA); CS/FG/BA/0.1CDs: CS/FG/BA film containing 0.1% carbon dots (CDs). (For interpretation of the references to color in this figure legend, the reader is referred to the web version of this article.)

As shown in Fig. 6A, the pH of large yellow croaker during refrigerated storage followed a typical “decline-rise” trend, a phenomenon widely reported in fish preservation studies (Kim et al., 2023). The initial pH was 6.4, and during the early stage (0–3 days), all samples showed a decrease, with the CK and CS/FG group exhibiting the sharpest drop to approximately 5.9 on day 3. This decrease was mainly attributed to postmortem glycolysis of glycogen to pyruvate, which was subsequently converted into lactic acid by lactate dehydrogenase, together with ATP degradation generating inorganic phosphates, leading to muscle acidification (Daskalova, 2019).

From day 4 onwards, the pH gradually increased. The CK and CS/FG group rose rapidly, reaching approximately 7.2 by day 12, significantly higher than the CS/FG/BA0.1CDs groups. This increase was attributed to microbial metabolism, which generated large amounts of alkaline compounds such as ammonia and amines, consistent with the well-established mechanism of late-stage spoilage in refrigerated fish (Abbas et al., 2008). By contrast, the CS/FG/BA0.1CDs group effectively suppressed pH increase, which showed a mild rise, reaching only 6.89 ± 0.11 on day 12. Such effects are likely associated with the antibacterial activity of CDs, which inhibit spoilage bacteria proliferation and consequently reduce proteolytic metabolism and the accumulation of alkaline by-products (Wang et al., 2024). These findings indicate a significant interaction between film type and storage time, as evidenced in **Table S2**. Specifically, the pH changes during storage were strongly influenced by the type of film applied, in which the CS/FG/BA/0.1CDs film resulted in a notably lower pH increase compared to the CK group over the storage period. Similarly, significant interaction effects were observed for other quality indicators, including TVB-N, TVC, TBARS, hardness, chewiness, drip loss, and sensory evaluation. In all cases, the CS/FG/BA/0.1CDs film contributed to more favorable quality attributes than the CK film throughout the storage period.

TVB–N, primarily composed of ammonia, trimethylamine, and other volatile amines, reflects the extent of microbial- and enzyme-mediated protein degradation. As shown in the Fig. 6B. At day 0, the TVB–N content of all groups was approximately 9 mg N/100 g, reflecting good initial quality. By day 6, the CK group had risen to 23.91 ± 2.64 mg N/100 g, approaching the spoilage threshold of 30 mg N/100 g (Fan et al., 2009), whereas the CS/FG/BA and CS/FG/BA/0.1CDs groups remained significantly lower at 16.63 ± 0.85 and 14.52 ± 1.01 mg N/100 g, respectively, indicating effective inhibition of protein degradation. During the later stage (days 9–12), TVB–N levels rose sharply. On day 12, the CK group reached 61.25 ± 1.92 mg N/100 g, far exceeding the acceptable limit, indicating severe spoilage. The CS/FG group also increased significantly to 51.75 ± 1.14 mg N/100 g. By contrast, the CS/FG/BA and CS/FG/BA/0.1CDs groups showed slower increases, reaching 35.7 ± 1.23 and 28.9 ± 1.44 mg N/100 g, respectively, both significantly lower than the CK and CS/FG group.

Consistently, TVC **(**Fig. 6C**)** increased with storage time. All groups started at ∼3.5 log CFU/g (first-grade freshness). CK exceeded 6 log CFU/g by day 9 (6.50 ± 0.01 log CFU/g) and reached 7.31 ± 0.06 log CFU/g by day 12. As 7.0 log CFU/g is the internationally recognized upper microbiological limit for fresh fish (Horwitz, 1975), this value indicated that the control samples had undergone severe spoilage. By contrast, CS/FG/BA and CS/FG/BA/0.1CDs remained below the inedibility threshold at day 12 (6.45 ± 0.07 and 6.11 ± 0.05 log CFU/g), confirming that the films effectively suppressed microbial proliferation and delayed protein degradation. In addition to proteolysis, lipid oxidation is another major spoilage pathway (Mozzon et al., 2024). Unlike TVB–N, which reflects protein degradation, lipid oxidation mainly affects sensory attributes such as flavor and color, and produces malondialdehyde (MDA), which is harmful to consumers (Xiong et al., 2021). As shown in Fig. 6D**,** at day 0, all groups exhibited similar values (∼0.45 mg MDA/kg), indicating minimal initial oxidation. On day 6, the CK and CS/FG group rose to 0.73 ± 0.05 mg/kg and 0.78 ± 0.01 mg/kg respectively, both significantly higher than CS/FG/BA (0.51 ± 0.02 mg/kg) and CS/FG/BA/0.1CDs (0.48 ± 0.01 mg/kg). On day 12, CK rapidly increased to 2.01 ± 0.28 mg/kg, surpassing the acceptable threshold of 1–2 mg /kg (Fan et al., 2009), whereas CS/FG/BA/0.1CDs remained at 0.69 ± 0.07 mg/kg, still within acceptable limits.

Textural attributes such as chewiness and hardness are jointly influenced by multiple biochemical and physical processes during storage, including protein denaturation, myofibrillar fragmentation, water loss, lipid oxidation, and microbial activity (Wu et al., 2018). As shown in Fig. 6E**&F**, both chewiness and hardness declined over time. For chewiness, all groups had similar initial values (∼0.49 mj) but declined during storage. The CK and CS/FG group decreased most rapidly, reaching 0.24 ± 0.015 and 0.26 ± 0.015 mj by day 12, whereas CS/FG/BA and CS/FG/BA/0.1CDs maintained significantly higher values of 0.3569 ± 0.015 and 0.3700 ± 0.010 mj, respectively. Hardness showed a similar trend: CK declined from 47.37 ± 0.61 g to 22.50 ± 1.25 g after 12 days of storage, CS/FG dropped to 25.20 ± 0.40 g, while CS/FG/BA and CS/FG/BA/0.1CDs remained higher at 32.20 ± 0.78 and 35.83 ± 0.93 g, respectively. Collectively, the CS/FG/BA/0.1CDs group maintained superior textural stability throughout storage.

#### 3.4.3 Drip loss and sensory evaluation

Drip loss is a key indicator for evaluating the water-holding capacity and structural integrity of aquatic products during storage or processing (Chan et al., 2022). Its variation reflects the stability of muscle proteins and the strength of water-protein interactions, thereby exerting influence on sensory attributes, flavor retention, and consumer acceptance (Nakazawa & Okazaki, 2020). As shown in Fig. 6G, the drip loss of large yellow croaker fillets exhibited an upward trend throughout refrigerated storage, reaching relatively high values on days 9–12, which indicates a progressive decline in water-holding capacity with prolonged storage. Significant differences among groups emerged in the late storage stages. On day 12, the drip loss of the CK and CS/FG groups rose to 15.17 ± 0.9% and 13.25 ± 0.75%, respectively. In contrast, the CS/FG/BA and CS/FG/BA/0.1CDs groups maintained much lower drip losses of 10.96 ± 0.18% and 10.18 ± 0.63%, respectively, both significantly lower than CK and CS/FG group.

Sensory evaluation provides a comprehensive assessment of fish freshness by integrating appearance, odor, texture, and color attributes (Zhang et al., 2022). As shown in Fig. 6H, all groups maintained high sensory scores during the early storage period (0–3 days), with fillets exhibiting bright surface color, firm and elastic texture, and no detectable off-odor (total score > 13, rated as “excellent”). As storage progressed, the CK and CS/FG groups showed sharp declines, dropping to 9.87 and 10.57 on day 9 and further down to 5.53 and 7.99 by day 12, characterized by dull color, reduced elasticity, and off-odor development. In contrast, the CS/FG/BA and CS/FG/BA/0.1CDs groups retained a significantly higher score of 9.80 and 10.19 by day 12 respectively, remaining above the “threshold of sensory rejection” range. Although sensory quality is shaped by multiple biochemical and physical processes, its preservation was generally consistent with the suppression of drip loss (Mozzon et al., 2024; Zhang et al., 2022), suggesting that improved water-holding capacity contributed to the more desirable appearance and texture in BA- and CD-containing films. Collectively, integrating pH, TVB–N, TBARS, TVC, texture, drip loss, and sensory data, it is evident that the combined incorporation of BA and CDs markedly enhanced the overall preservation performance of CS/FG films. During refrigerated storage, the CS/FG/BA/0.1CDs packaging most effectively inhibited pH rise, suppressed microbial growth, and delayed the accumulation of TVB–N and TBARS, demonstrating its efficacy in retarding protein and lipid degradation. Simultaneously, it maintained structural stability, minimized water loss, and preserved sensory quality, particularly in the later storage stages, effectively mitigating the overall quality deterioration of the fillets. These findings highlight the synergistic effects of BA and CDs, which not only prolonged the shelf life of refrigerated large yellow croaker fillets but also enabled real-time freshness monitoring through a sensitive colorimetric response. Consequently, CS/FG/BA/0.1CDs films serve as advanced, multifunctional packaging materials for high-value aquatic products.

## 4 Conclusion

In this study, multifunctional composite films were successfully developed by incorporating blueberry anthocyanins (BA) and carbon dots (CDs) derived from *Porphyra haitanensis* residues into a chitosan/fish gelatin (CS/FG) matrix. The addition of BA and 0.1% CDs markedly enhanced the structural and functional attributes of the films, including improved thermal stability, UV shielding, antioxidant activity, and antibacterial efficacy, while maintaining balanced mechanical strength and barrier properties. This multi-dimensional improvement is attributed to the synergistic cross-linking and reinforcement provided by the BA and CDs within the biopolymer network. The CS/FG/BA/0.1CDs film also demonstrated stable and sensitive colorimetric responses to both pH variations and ammonia vapor, offering an active and intelligent system for real-time food monitoring. When applied to the preservation of large yellow croaker fillets under refrigerated storage, the film effectively suppressed pH elevation, delayed the accumulation of total volatile basic nitrogen (TVB–N) and thiobarbituric acid-reactive substances (TBARS), inhibited microbial growth, and preserved textural integrity, drip loss, and sensory quality by reinforcing the physical barrier and providing sustained antioxidant and antimicrobial activities. These findings highlight the synergistic functionalities of BA and CDs, which not only prolonged the shelf life of the fillets by approximately 6 days but also established a direct correlation between the film's colorimetric transition and the biochemical spoilage stages. Consequently, the CS/FG/BA/0.1CDs films serve as advanced, multifunctional packaging materials that integrate both preservation and intelligent sensing for high-value aquatic products. Future research should focus on advancing the sustainable synthesis of CDs from *Porphyra haitanensis* residues, with an emphasis on environmental sustainability, as well as exploring the scalability of this approach. Overall, this work demonstrates a feasible route to valorize marine biomass residue by converting it into functional carbon dots and illustrates their potential use as an additive for sustainable, intelligent food packaging materials aligned with circular bioeconomy principles.

## CRediT authorship contribution statement

**Zijia Zhan:** Writing – original draft, Visualization, Software, Data curation, Conceptualization. **Yi Guan:** Writing – review & editing, Visualization, Software, Conceptualization. **Can Guo:** Visualization, Conceptualization. **Junchao Huang:** Visualization, Software, Data curation. **Huawei Zheng:** Visualization, Software. **Fude Liang:** Visualization, Software. **Zhiyu Li:** Visualization, Conceptualization. **Quan (Sophia) He:** Funding acquisition, Data curation. **Yijing Wu:** Resources. **Qinshan Huang:** Visualization, Software. **Jie Yang:** Writing – review & editing, Software, Funding acquisition.

## Declaration of competing interest

The authors declare that they have no known competing financial interests or personal relationships that could have appeared to influence the work reported in this paper.

## Data Availability

The authors do not have permission to share data.

## Appendix A Supplementary data

Supplementary material Image 1
