# Corrigendum to “Seaweed residue–derived carbon dots composite films for spoilage-responsive monitoring and preservation of large yellow croaker fillets” [Food Chem. X 34 (2026) 103626]

**DOI:** 10.1016/j.fochx.2026.103760

**Published:** 2026-03-22

**Authors:** Zijia Zhan, Yi Guan, Can Guo, Junchao Huang, Huawei Zheng, Fude Liang, Zhiyu Li, Quan (Sophia) He, Yijing Wu, Qinshan Huang, Jie Yang

**Affiliations:** a Fuzhou Institute of Oceanography, College of Geography and Oceanography, Minjiang University, Fuzhou, China; b Fujian Key Laboratory of Marine Enzyme Engineering, College of Biological Science and Engineering, Fuzhou University, Fuzhou, China; c Department of Orthopedics, Fujian Orthopaedics Research Institute, the First Affiliated Hospital of Fujian Medical University, Fuzhou, China; d Department of Orthopedics, National Regional Medical Center, Binhai Campus of the First Affiliated Hospital, Fujian Medical University, Fuzhou, China; e Fujian Key Laboratory on Conservation and Sustainable Utilization of Marine Biodiversity, Minjiang University, Fuzhou, China; f Department of Engineering, Faculty of Agriculture, Dalhousie University, Truro, NS, Canada

The authors regret that they have noticed an error in the affiliation details.

The affiliation "a" is currently listed as the Institute of Oceanography. However, the correct affiliation should be the Fuzhou Institute of Oceanography. The correct affiliation is provided below:

a Fuzhou Institute of Oceanography, College of Geography and Oceanography, Minjiang University, Fuzhou, China

The affiliation listed in this corrigendum is correct and final.

The authors would like to apologise for any inconvenience caused.

