# Supplementary material for: Seaweed residue–derived carbon dots composite films for spoilage-responsive monitoring and preservation of large yellow croaker fillets
Source: Food Chem X. 2026 Feb 3;34:103626. doi: 10.1016/j.fochx.2026.103626 (PMC12907725; doi:10.1016/j.fochx.2026.103626)
Supplement: Supplementary file 1 — Supplementary material [file mmc1.docx]

**Supplemental Materials**

**for**

**Seaweed residue–derived carbon dots composite films for spoilage-responsive monitoring and preservation of large yellow croaker fillets**

Zijia Zhan^a,b,1^, Yi Guan^b,1^, Can Guo^a^, Junchao Huang^a^, Huawei Zheng^a,e^, Fude Liang^c,d^, Zhiyu Li^a,e^, Quan (Sophia) He^f^, Yijing Wu^a,e,*^, Qinshan Huang^c,d,*^, Jie Yang^a,e,*^

a Institute of Oceanography, College of Geography and Oceanography, Minjiang University, Fuzhou, China

b The Key Laboratory of Novel Enzyme Design and Creation of Fujian Province, Fuzhou University, Fuzhou, China

c Department of Orthopedics, Fujian Orthopaedics Research Institute, the First Affiliated Hospital of Fujian Medical University, Fuzhou, China

d Department of Orthopedics, National Regional Medical Center, Binhai Campus of the First Affiliated Hospital, Fujian Medical University, Fuzhou, China

e Fujian Key Laboratory on Conservation and Sustainable Utilization of Marine Biodiversity, Minjiang University, Fuzhou, China

f Department of Engineering, Faculty of Agriculture, Dalhousie University, Truro, NS, Canada

**Table S1.** Sensory evaluation criteria for chilled large yellow croaker fillets (15-point scale).

| **Attribute** | **Excellent (13–15)** | **Good (10–12)** | **Acceptable (6–9)** | **Unacceptable (0–5)** |
| --- | --- | --- | --- | --- |
| **Color** | Bright and fresh; flesh color clear; muscle texture distinct and translucent | Relatively bright; flesh slightly dull; texture still clear | Pale or dull; texture blurred | Darkened or discolored; texture severely blurred; sticky |
| **Odor** | Typical fresh fish odor; fresh and pleasant; no off-odor | Odor relatively fresh; slight stale odor | Noticeable off-odor; slight spoilage smell | Obvious off-odor; sour or putrid smell |
| **Tissue status** | Muscle fibers compact and firm; texture smooth | Muscle fibers compact; texture relatively smooth | Muscle fibers less compact, not loose; texture relatively rough | Muscle fibers loose or mushy; texture unclear |
| **Elasticity** | Firm with good elasticity; finger indentation disappears immediately | Firm with elasticity; finger indentation disappears quickly | Moderate elasticity; finger indentation disappears slowly | No elasticity; finger indentation remains |

**Table S2** ANOVA for pH, TVB-N, TVC, TBARS, hardness, chewiness, drip loss and sensory evaluation for different types of film under storage.

|  | **Type of film** | | **Storage time** | | **Interaction** | |
| --- | --- | --- | --- | --- | --- | --- |
|  | F-value | p-value | F-value | p-value | F-value | p-value |
| **pH** | 3.32 | 0.029 | 376.86 | 0.000 | 6.89 | 0.000 |
| **TVB-N** | 242.17 | 0.000 | 1268.28 | 0.000 | 57.19 | 0.000 |
| **TVC** | 411.76 | 0.000 | 3845.49 | 0.000 | 58.58 | 0.000 |
| **TBARS** | 144.16 | 0.000 | 242.88 | 0.000 | 32.11 | 0.000 |
| **Hardness** | 279.88 | 0.000 | 956.65 | 0.000 | 25.28 | 0.000 |
| **Chewiness** | 77.54 | 0.000 | 278.53 | 0.000 | 11.72 | 0.000 |
| **Drip loss** | 51.22 | 0.000 | 2245.91 | 0.000 | 21.54 | 0.000 |
| **Sensory score** | 41.47 | 0.000 | 561.60 | 0.000 | 21.53 | 0.000 |

Note: Total volatile basic nitrogen (TVB-N); Total viable counts (TVC); Thiobarbituric acid reactive substances (TBARS). p-value < 0.05 indicates significant influence.

**Section S2. Additional Figures**


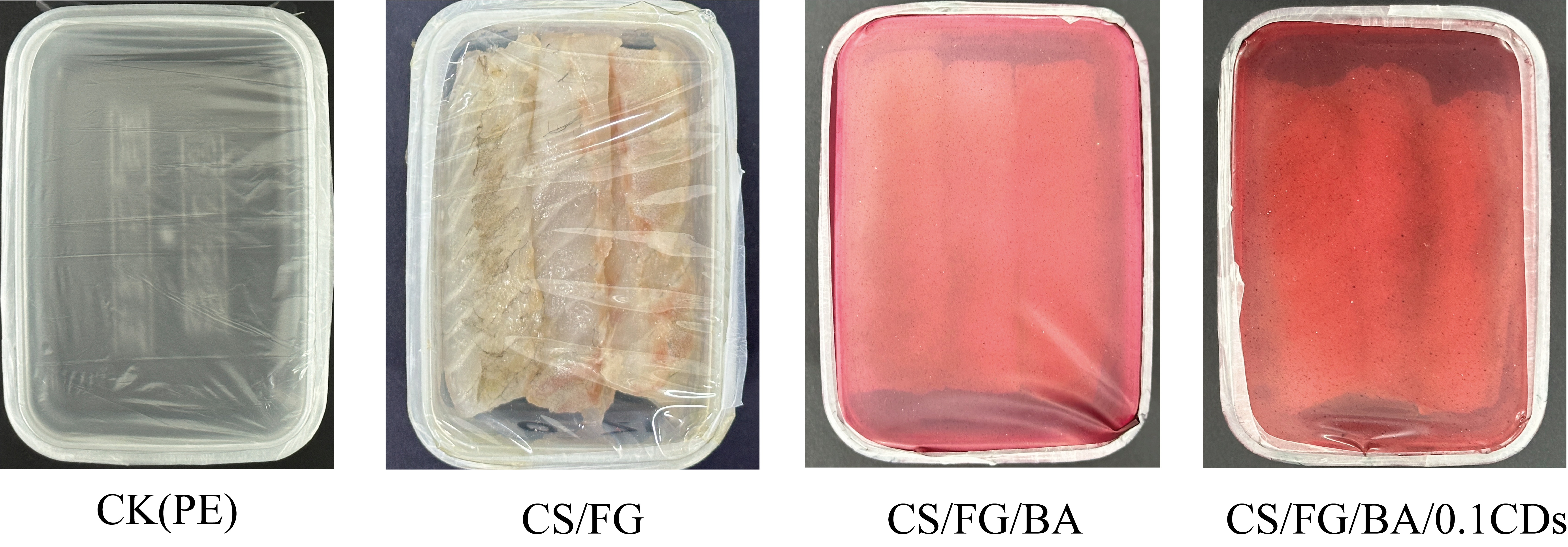


**Fig. S1** Packaging setup showing fish fillets in a PP box sealed with the functional film and secured by Parafilm.

**
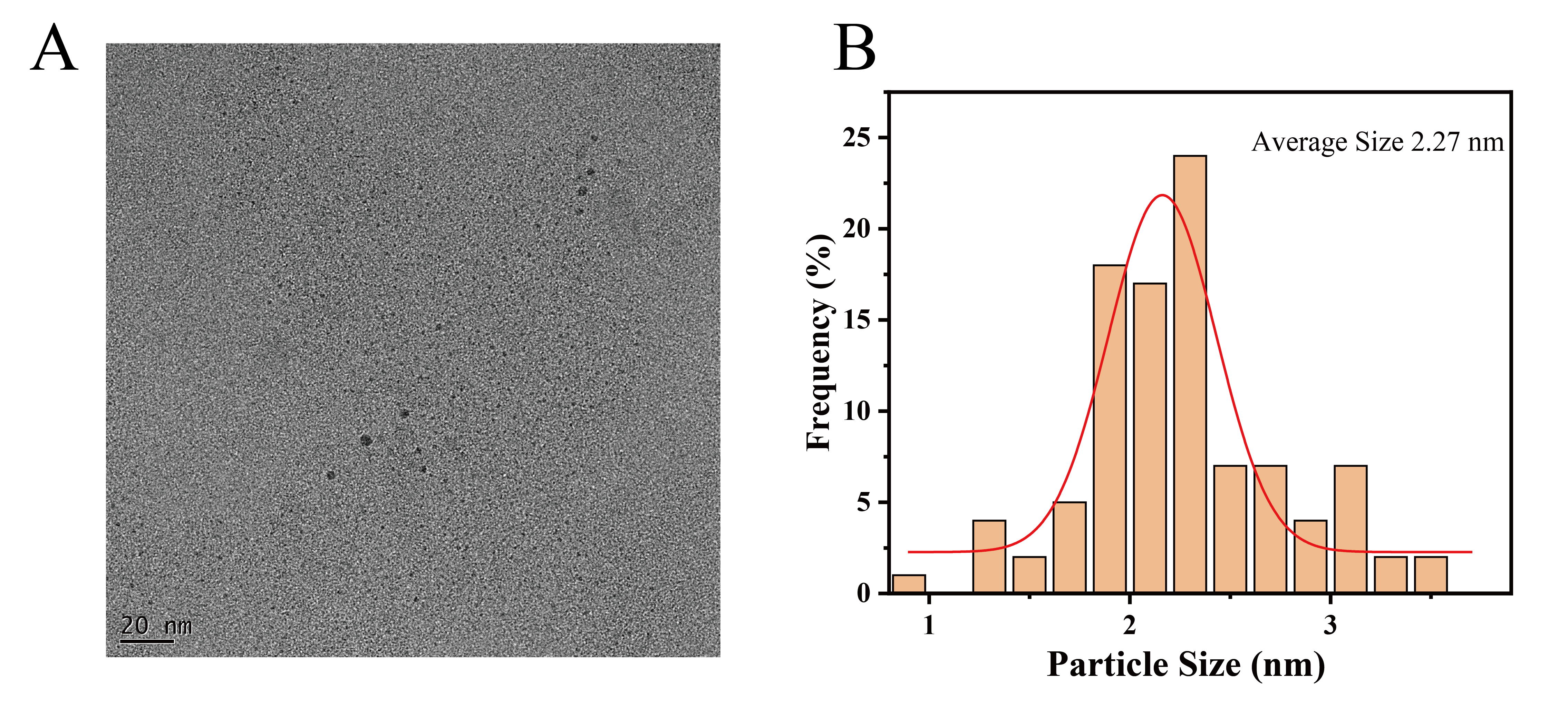
**

**Fig. S2** (A) Transmission electron microscopy (TEM) image of carbon dots (CDs) derived from *Porphyra haitanensis* residues; scale bar: 5 nm. (B) Diameter distribution of carbon dots (CDs).


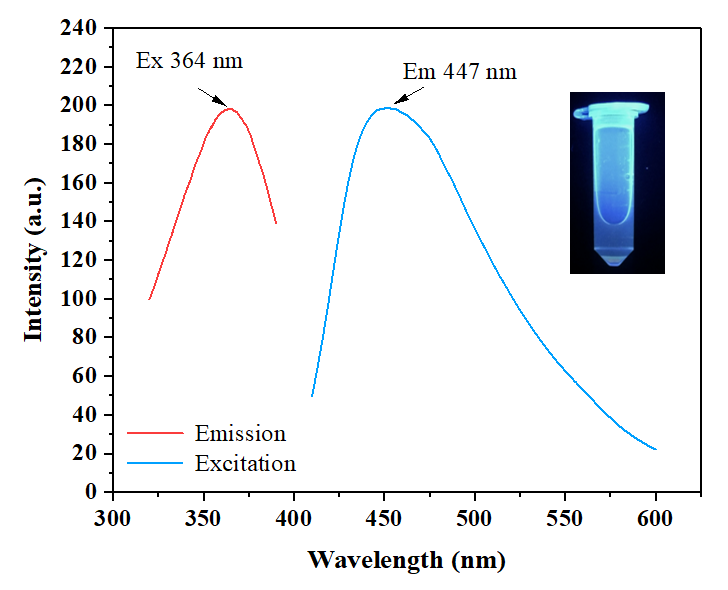


**Fig. S3** Fluorescence emission, and excitation spectra of carbon dots (CDs).


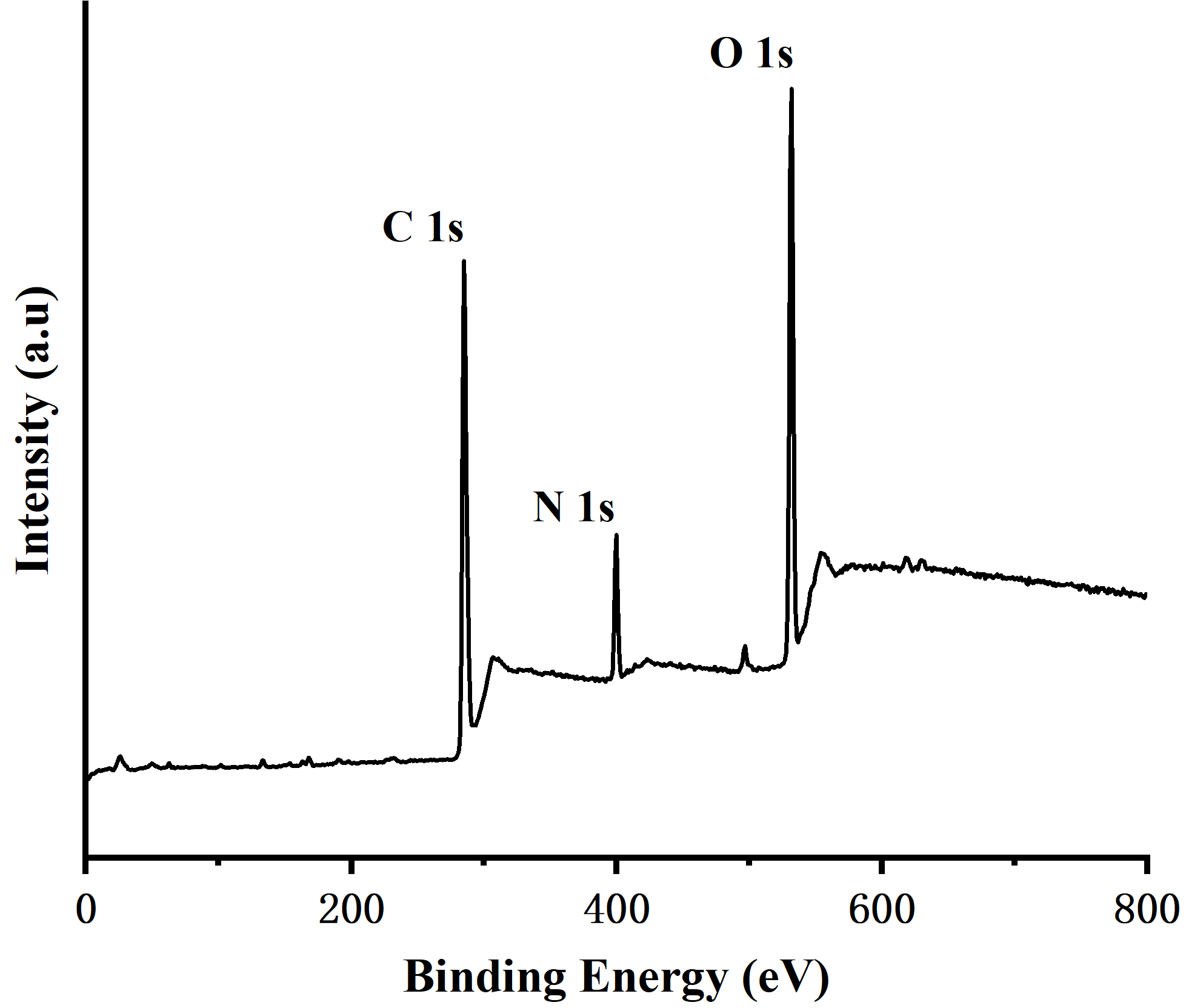


**Fig. S4** X-ray photoelectron spectroscopy survey scan, high-resolution of carbon dots (CDs).

**Fig. S5** Thermogravimetric Analysis (TG) of different films. Film formulations: CS/FG: chitosan/fish gelatin film; CS/FG/BA: chitosan/fish gelatin film containing blueberry anthocyanins (BA); CS/FG/BA/0.1CDs: CS/FG/BA film containing 0.1% carbon dots (CDs); CS/FG/BA/0.5CDs and CS/FG/BA/1.0CDs: CS/FG/BA films containing 0.5% and 1.0% CDs, respectively.


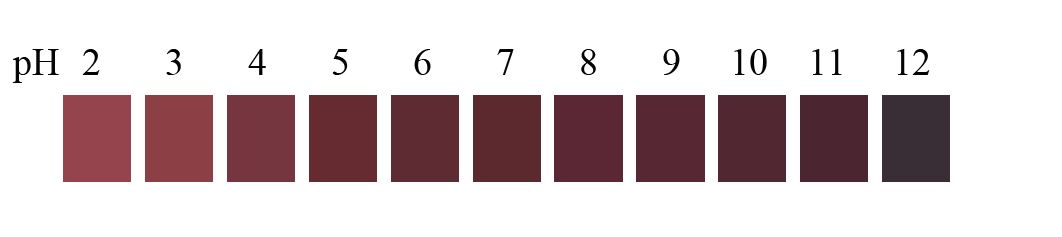


**Fig. S6** Color change of CS/FG/BA (chitosan/fish gelatin film incorporated with blueberry anthocyanins) in different pH solutions.

**
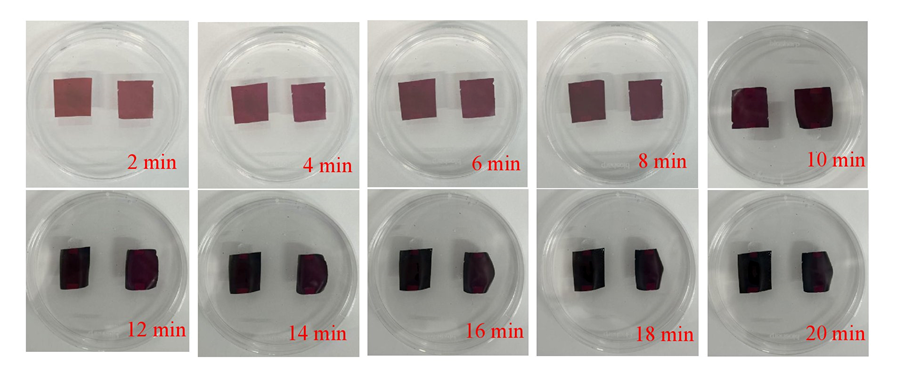
**

**Fig. S7** Color response of CS/FG/BA (chitosan/fish gelatin film containing blueberry anthocyanins) to ammonia (NH3) vapor.
